# Association between miR-146a and Tumor Necrosis Factor Alpha (TNF-α) in Stable Coronary Artery Disease

**DOI:** 10.3390/medicina57060575

**Published:** 2021-06-04

**Authors:** Tiago Pereira-da-Silva, Patrícia Napoleão, Marina C. Costa, André F. Gabriel, Mafalda Selas, Filipa Silva, Francisco J. Enguita, Rui Cruz Ferreira, Miguel Mota Carmo

**Affiliations:** 1 Department of Cardiology, Hospital de Santa Marta, Centro Hospitalar Universitário de Lisboa Central, 1169-024 Lisbon, Portugal; mafalda.selas@gmail.com (M.S.); felipafernandes@gmail.com (F.S.); cruzferreira@netcabo.pt (R.C.F.); 2 NOVA Doctoral School, Faculdade de Ciências Médicas, Universidade NOVA de Lisboa, 1169-056 Lisbon, Portugal; 3 Instituto de Medicina Molecular João Lobo Antunes, Universidade de Lisboa, 1649-028 Lisbon, Portugal; napoleao.patricia@gmail.com (P.N.); marinacosta@medicina.ulisboa.pt (M.C.C.); andre.gabriel@medicina.ulisboa.pt (A.F.G.); fenguita@medicina.ulisboa.pt (F.J.E.); 4 Cardiomics Unit, Centro Cardiovascular da Universidade de Lisboa, Faculdade de Medicina, Universidade de Lisboa, 1649-028 Lisbon, Portugal; 5 Chronic Diseases Research Center (CEDOC), NOVA Medical School, Faculdade de Ciências Médicas, Universidade NOVA de Lisboa, 1150-082 Lisbon, Portugal; mabmc@sapo.pt

**Keywords:** inflammation, microRNA, miR-146a, stable coronary artery disease, tumor necrosis factor alpha

## Abstract

*Background and Objectives*: Tumor necrosis factor alpha (TNF-α) is proatherogenic and associated with the risk of acute ischemic events, although the mechanisms that regulate TNF-α expression in stable coronary artery disease (SCAD) are not fully understood. We investigated whether metabolic, inflammatory, and epigenetic (microRNA (miRNA)) markers are associated with TNF-α expression in SCAD. *Materials and Methods*: Patients with SCAD were prospectively recruited and their metabolic and inflammatory profiles were assessed. TNF-α levels were assessed using an enzyme-linked immunosorbent assay. The relative expression of six circulating miRNAs associated with the regulation of inflammation and/or atherosclerosis was determined. *Results*: Of the 24 included patients with the mean age of 65 (9) years, 88% were male, and 54% were diabetic. The TNF-α levels were (median (interquartile range)) 1.0 (0.7–1.1) pg/mL. The percentage of glycosylated hemoglobin (*r* = 0.418, *p* = 0.042), serum triglyceride levels (*r* = 0.429, *p* = 0.037), and C-reactive protein levels (*r* = 0.407, *p* = 0.048) were positively correlated with TNF-α levels. Of the candidate miRNAs, miR-146a expression levels were negatively correlated with TNF-α levels (as indicated by *r* = 0.500, *p* = 0.035 for correlation between delta cycle threshold (ΔC_t_) miR-146a and TNF-α levels). In multivariate analysis, serum triglyceride levels and miR-146a expression levels were independently associated with TNF-α levels. miR-146 expression levels were not associated with metabolic or other inflammatory parameters and were negatively correlated with the number of coronary vessels with obstructive disease (as indicated by *r* = 0.556, *p* = 0.017 for correlation between ΔC_t_ miR-146a and number of diseased vessels). *Conclusions*: miR-146a expression levels were negatively correlated with TNF-α levels in patients with SCAD, irrespective of other metabolic or inflammatory markers, and with the severity of coronary artery disease. The results add to the knowledge on the role of miR-146a in TNF-α-based inflammation in SCAD and support future research on the potential therapeutic use of miR-146a in such a clinical scenario.

## 1. Introduction

Stable coronary artery disease (SCAD) is accountable for substantial cardiovascular morbidity and mortality worldwide [1]. It is a chronic inflammatory syndrome characterized by the activation of proinflammatory pathways, including those of tumor necrosis factor alpha (TNF-α), soluble CD40 ligand, and C-reactive protein [2,3,4]. Specifically, TNF-α is a strong proinflammatory mediator that induces atherogenesis [2,5]. Moreover, TNF-α is closely associated with cardiovascular prognosis and increased TNF-α levels are associated with a higher risk of ischemic events [6,7]. Therefore, TNF-α has been studied as a therapeutic target in patients with documented atherosclerosis [8,9,10].

Despite the acknowledged role of TNF-α in atherosclerosis development and progression, the mechanisms that regulate TNF-α expression in patients with SCAD are not fully understood [5]. Metabolic dysregulation, including hyperglycemia, dyslipidemia, and adiposity, increases TNF-α levels [11,12,13,14,15,16]. Such metabolic abnormalities interact with each other and often coexist in patients with SCAD, and the independent role of each on TNF-α expression is not entirely known [5,17]. On the other hand, epigenetic modulators, including microRNAs (miRNAs), may influence TNF-α expression in vitro [18]. miRNAs are small noncoding RNA molecules that are involved in distinct biological roles, including the regulation of inflammation and atherosclerosis [19]. Whether an interaction between circulating miRNAs and TNF-α expression exists in patients with SCAD and whether such potential association is influenced by metabolic abnormalities or coexistent inflammatory dysregulation is unknown. The knowledge on the expression of inflammatory mediators, including TNF-α, and their regulators in SCAD may provide insights into its pathophysiology. Such research area has regained interest after the clinical results of a pure anti-inflammatory agent in patients with stable atherosclerosis [20].

We investigated whether metabolic, inflammatory, and epigenetic (miRNA) markers are associated with TNF-α expression in SCAD.

## 2. Materials and Methods

This study is a part of a project aimed at assessing inflammatory and epigenetic signatures associated with the clinical expression of stable atherosclerosis. The study protocol was approved by the ethics committees of the involved institutions (Centro Hospitalar Universitário de Lisboa Central, Nr. 245/2015, 1 October 2015; and NOVA Medical School | Faculdade de Ciências Médicas, Universidade NOVA de Lisboa, Nr. 000176, 11 November 2015). The investigation conformed to the principles outlined in the Helsinki Declaration. All the participants signed informed consent forms.

### 2.1. Recruitment of Participants

Patients with SCAD from our center were prospectively recruited. The inclusion criteria were the presence of coronary artery disease, defined as luminal stenosis of at least 50% for the left main artery or at least 70% for other epicardial vessels on invasive coronary angiography, and absence of symptoms worsening in the prior six months. Patients with acute ischemic events within 12 months in any arterial territory, those with coronary artery bypass grafting performed within 12 months, those with prior percutaneous arterial treatment, those with heart failure, hemodynamically significant valvular heart disease, hematological disorders, active infection, history of malignancy, chronic kidney disease (stage 4 or 5), or severe hepatic dysfunction, those under 18 years of age, and those unable or unwilling to consent to participate in the study were excluded.

### 2.2. Data Collection and Blood Sampling

Data were collected prospectively after patient inclusion. A standardized record including clinical, demographic, laboratory, echocardiographic, and angiographic data was obtained from each participant. Peripheral blood was collected early in the morning under fasting conditions before any coronary intervention. Serum was separated by centrifugation (500× *g* for 10 min) within 15 min of sampling. Aliquots were stored at −80 °C and samples were thawed only once.

### 2.3. Measurements of Inflammatory Markers

Levels of TNF-α, soluble CD40 ligand, and C-reactive protein were measured in serum by an enzyme-linked immunosorbent assay (R&D Systems, Minneapolis, MN, USA). Measurements were performed in duplicates. The intra-assay variation among the duplicates for all samples was less than 10%.

### 2.4. Quantification of Expression Levels of Candidate miRNAs

Six candidate miRNAs (miR-21, miR-27b, miR-29a, miR-126, miR-146a, and miR-218) were selected based on the following criteria: miRNAs are associated with the regulation of inflammation and/or atherosclerosis in experimental models [19,21,22]; each of the miRNAs regulates distinct pathways and/or has distinct mechanisms of action [19,21,22]; and miRNAs were reported to be dysregulated in patients with stable atherosclerosis [23,24,25,26,27,28,29]. Among other functions, miR-21 regulates vascular smooth cell and endothelial cell functions; miR-27b regulates lipid metabolism and development of lipid-induced atherosclerotic lesions, and modulates the production of proinflammatory factors; miR-29a regulates fibrosis and extracellular matrix composition; miR-126 regulates endothelial function in response to shear stress; miR-146a regulates endothelial and monocyte-macrophage inflammatory response; and miR-218 regulates endothelial cell migration [18,19,21,22].

Total RNA was extracted from serum samples using the miRCURY™ RNA Isolation Kit (Qiagen, Hilden, Germany). Complementary DNA was synthesized from total RNA using the Universal cDNA synthesis kit from miRCURY™ LNA miRNA system (Qiagen, Hilden, Germany). miRNA amplification was performed using quantitative reverse-transcription polymerase chain reaction (using the miRCURY™ LNA SYBR Green PCR Kit and LNA™ PCR primers, Qiagen, Hilden, Germany), and the melting curve was determined according to the following conditions: 95 °C for 10 min followed by 45 cycles of 95 °C for 10 s and 60 °C for 60 s. All the reactions were performed in triplicates. The amplification data were assessed using DataAssist™ Software v3.01 (Thermo Fisher Scientific, Waltham, MA, USA). Cycle threshold (C_t_) values greater than 40 were considered undetermined [25,30,31,32]. The relative expression levels of the six candidate miRNAs were calculated using the delta cycle threshold (ΔC_t_) method, normalizing for the UniSp6 RNA spike-in control [25,33,34,35]. Higher ΔC_t_ values represent lower circulating levels of the candidate miRNAs [25,33,34,35].

### 2.5. Statistical Analysis

Discrete variables are presented as frequencies (percentages) and continuous variables are presented as the mean (standard deviation) in normally distributed data or median (interquartile range) in variables without a normal distribution (Shapiro–Wilk test). Categorical variables were analyzed using the chi-squared or Fisher’s exact tests. Continuous variables were analyzed using Student’s t-test or the Mann–Whitney test when normality was not verified. Pearson’s correlation was used to test correlations between continuous variables. A multivariate linear regression analysis was performed using TNF-α as the dependent variable and the metabolic, inflammatory, and epigenetic (miRNA) parameters associated with TNF-α in the univariate analysis as the independent variables. Variables with a *p*-value < 0.10 in the univariate analysis were tested in the multivariate model. A correction for collinearity was performed. The level of significance was set at α = 0.05. Analyses were conducted using SPSS software, version 26.0 (IBM Corp, Armonk, NY, USA).

## 3. Results

### 3.1. Clinical Characteristics and Laboratory Data Of Patients

A total of 24 participants were recruited with a mean age of 65 (9) years, of which 21 (88%) were male and 13 (54%) were diabetic (Table 1). All patients were using single antiplatelet therapy and lipid-lowering (mostly statin) therapy. No modifications in antithrombotic or lipid-lowering therapy were made within the two months prior to blood sampling. The median (interquartile range) TNF-α levels were 1.0 (0.7–1.1) pg/mL.

### 3.2. Parameters Associated with TNF-α Levels in Univariate Analysis

The parameters associated with TNF-α levels in the univariate analysis are presented in Table 2. The percentage of glycosylated hemoglobin, serum triglyceride levels, and C-reactive protein levels were positively correlated with TNF-α levels, and there was a trend for a positive correlation between fasting glycemia and TNF-α levels. No associations were found between other clinical parameters (including pharmacological therapy) or other laboratory data (including soluble CD40 ligand levels) and TNF-α levels.

Regarding the candidate miRNAs, ΔC_t_ miR-146a showed a positive correlation with TNF-α levels (*r* = 0.500, *p* = 0.035; Table 2), indicating an inverse correlation between miR-146a expression levels and TNF-α levels. The expression levels of other miRNAs were not associated with TNF-α levels.

### 3.3. Parameters Associated with TNF-α Levels in Multivariate Analysis

In the multivariate analysis, serum triglyceride levels and miR-146a expression levels were independently associated with TNF-α levels. Lower miR-146a expression levels and higher serum triglyceride levels were associated with increased TNF-α levels (Table 3).

### 3.4. MiR-146a Expression in Patients with Stable Coronary Artery Disease

miR-146a expression levels were not associated with other metabolic or inflammatory parameters, including the percentage of glycosylated hemoglobin, serum triglyceride levels, or C-reactive protein levels (Supplementary Material, Table S1). There was a positive correlation between ΔC_t_ miR-146a and the number of coronary vessels with obstructive disease (*r* = 0.556, *p* = 0.017), indicating lower expression levels of miR-146a in association with a higher severity of coronary artery disease.

## 4. Discussion

In this prospective study, three main findings were noted: in patients with SCAD, metabolic and epigenetic (miRNA) mediators were associated with TNF-α expression; miR-146a expression levels were negatively correlated with TNF-α levels, irrespective of other metabolic and inflammatory parameters; and miR-146a expression levels were negatively correlated with the severity of coronary artery disease.

Identifying major inflammatory regulators in SCAD is relevant for providing insights into its pathophysiology as well as for clinical practice, since such regulators may be useful as biomarkers and possible therapeutic targets [20,36]. TNF-α is a proinflammatory agent with a distinct mechanism of action compared with other inflammatory mediators, which reinforces the relevance of clinical investigation addressing TNF-α regulation in atherosclerosis [5]. Moreover, TNF-α is a proatherogenic agent and closely associated with prognosis in patients with coronary artery disease [2,6,7]. Different anti-TNF-α therapies have been studied in patients with documented atherosclerosis, although the clinical results were not entirely consistent [8,9,10] and further insights are warranted regarding the regulation of TNF-α in patients with SCAD.

In this study, metabolic dysregulation, characterized by a higher percentage of glycosylated hemoglobin or higher serum triglyceride levels, was associated with higher TNF-α levels, which is consistent with the reports from preclinical models [11,12,13,14,15,16]. Of note, TNF-α may itself promote hyperglycemia and dyslipidemia, thereby further increasing cardiovascular risk [37,38]. Data on the association between epigenetic mediators and TNF-α expression are scarce. miR-146a was reported to suppress the inflammatory response, including TNF-α expression, by dampening the NF-κB pathway through interleukin 1 receptor-associated kinase, in experimental models [18,39]. In humans, a negative correlation between miR-146a expression levels and TNF-α levels has been described in patients with noncardiac inflammatory diseases, although such an association has not been described in patients with SCAD [40,41]. Consistently, we observed a negative correlation between miR-146a expression levels and TNF-α levels. Of note, such association was independent of other metabolic and inflammatory parameters, which are frequently abnormal in SCAD and influence TNF-α expression [11,12,13,14,15,16,17]. Moreover, the association between miR-146a expression levels and TNF-α levels was independent of other clinical parameters, including the pharmacological therapy, as demonstrated in uni- and multivariate analyses. These results support an independent role of miR-146a in the regulation of TNF-α-induced inflammation in SCAD.

We observed that miR-146a was negatively correlated with the severity of coronary artery disease. These results are consistent with data previously reported by our research group regarding the association between lower miR-146a expression levels and the extent of atherosclerosis to multiple arterial territories and higher severity of atherosclerosis in different territories [42]. Complementary atheroprotective and anti-inflammatory effects of miR-146 have been reported in experimental studies [19,21,43,44,45], which support the association between miR-146a expression levels and severity of coronary artery disease observed in this study. miR-146 is induced in endothelial cells in response to proinflammatory cytokines and acts as a negative feedback regulator of inflammatory signaling in endothelial cells by dampening the activation of proinflammatory transcriptional programs, including the NF-κB (as aforementioned), AP-1, and MAPK/EGR pathways, and by promoting eNOS expression [19,21,43]. The enhancement of miR-146 levels in the monocyte-macrophage lineage was also shown to suppress the NF-κB pathway and thus reduce macrophage activity [44]. Moreover, miR-146 targets the Toll-like receptor 4, reducing the formation of foam cells [45]. Therefore, miR-146 contributes to reduce vascular inflammation and atherosclerosis by targeting endothelial cells and the monocyte–macrophage lineage. The interaction between miR-146a and TNF-α [39] may be particularly relevant in such context.

The results of this study support the role of miR-146a in inflammation and atherosclerosis. Our findings and reported experimental data [18,19,21,39,43,44,45] suggest that miR-146a may be further investigated as a therapeutic target, complementary to other disease-modifying strategies, such as glycemic and lipid control. Specifically, the use of miR-146 mimics in patients with SCAD presenting enhanced inflammatory activation based on TNF-α levels may be a potential field of research [46]. Interestingly, lower miR-146a expression levels were reported to be associated with no response to an anti-inflammatory therapy in patients with COVID-19 and worse adverse outcomes [47]. This reinforces miR-146 as a potential target in experimental models of SCAD, considering the key role of inflammation in the regulation of atherosclerosis, particularly the TNF-α pathway.

There are strengths of this study that should be acknowledged. As far as we know, this is the first report describing the association between miR-146a expression levels and TNF-α levels in patients with SCAD. On the other hand, the multivariate analysis carried out increased the consistency of results by pointing to an independent association between miR-146a and TNF-α, irrespective of other metabolic and inflammatory parameters, which are frequently dysregulated in SCAD and influence TNF-α levels [11,12,13,14,15,16,17]. Moreover, the association between miR-146a and coronary artery disease severity added further to the consistency of the results. Finally, the results are in line with those from the experimental investigation [18].

### Study Limitations

The sample size was small and the results should be interpreted with caution. Nevertheless, the sample size allowed to detect a significant association between miR-146a expression levels and TNF-α levels and to adjust for confounders, which supports the validity of the results. Furthermore, the results are consistent with the preclinical data [18]. Of note, the sample included mostly male patients and the results may not be applicable to female patients. Larger prospective studies including a higher proportion of females are warranted for performing an external validation. On the other hand, a causal effect between miR-146a and TNF-α cannot be established based on the results of this study. Nevertheless, the adjustment for confounders in the multivariate analysis and the consistency of the results with the aforementioned experimental data [18] suggest that miR-146a is likely a regulator of TNF-α expression in patients with SCAD.

## 5. Conclusions

Metabolic and epigenetic (miR-146a) mediators were associated with TNF-α expression in patients with SCAD. miR-146a expression levels were negatively correlated with TNF-α levels, irrespective of other metabolic or inflammatory parameters, and with the severity of coronary artery disease. The results provide insights into the pathophysiology of inflammation in stable atherosclerosis, particularly the role of miR-146a in TNF-α-based inflammation in SCAD, and support future research on the potential therapeutic use of miR-146a in such a clinical scenario.

## Figures and Tables

**Table 1:** Clinical characteristics and laboratory data.

Clinical Characteristics	
Age, years	65 (9)
Male, *n* (%)	21 (88)
Hypertension, *n* (%)	22 (92)
Dyslipidemia, *n* (%)	22 (92)
Diabetes mellitus, *n* (%)	13 (54)
Active smoking, *n* (%)	4 (17)
Body mass index, kg/m^2^	26.7 (3.1)
Number of coronary vessels with obstructive disease ^1^	3 (2–4)
Prior coronary artery bypass grafting, *n* (%)	3 (13)
Left ventricular ejection fraction > 50%, *n* (%)	24 (100)
Antiplatelet therapy, *n* (%) ^2^	24 (100)
Oral anticoagulation, *n* (%)	0 (0)
Statin therapy, *n* (%)	22 (92)
High-intensity statin therapy, *n* (%)	17 (71)
Ezetimibe, *n* (%)	4 (17)
ACE inhibitor or ARB, *n* (%)	21 (88)
Betablocker, *n* (%)	15 (63)
Other antianginal agent, *n* (%)	12 (50)
Oral antidiabetic agent, *n* (%)	13 (54)
Insulin therapy, *n* (%)	6 (25)
**Laboratory Data**	
Hemoglobin, g/dL	13.6 (1.6)
Leukocyte count, 10^9^/L	8.0 (1.5)
Neutrophil count, 10^9^/L	4.7 (1.5)
Lymphocyte count, 10^9^/L	2.2 (1.7–2.7)
Platelet count, 10^9^/L	236 (41)
Fasting glycaemia, mg/dL	99 (85–166)
Percentage of glycosylated hemoglobin	6.1 (5.6–7.9)
Creatinine, mg/dL	0.9 (0.8–1.3)
Total cholesterol, mg/dL	158 (40)
LDL-cholesterol, mg/dL	92 (29)
HDL-cholesterol, mg/dL	35 (29–43)
Triglycerides, mg/dL	117 (87–162)
Soluble CD40 ligand, ng/mL	8.4 (2.5)
C-reactive protein, mg/L	4.0 (3.6–4.7)
**ΔC_t_ miRNA**	
miR-21	17.6 (3.2)
miR-27b	23.4 (20.9–24.4)
miR-29a	22.9 (2.8)
miR-126	23.1 (16.9–24.9)
miR-146a	22.5 (2.5)
miR-218	20.9 (19.5–24.3)

^1^ The left main artery, left anterior descending artery, circumflex artery, and right coronary artery were scored individually. Categorical variables are expressed as frequency (percentage) and continuous variables as the mean (standard deviation) or median (interquartile range); ^2^ all patients were using single antiplatelet therapy. ACE—angiotensin-converting enzyme; ARB—angiotensin II receptor blocker; HDL—high-density lipoproteins; LDL—low-density lipoproteins; miRNA—microRNA; ΔC_t_—delta cycle threshold.

**Table 2:** Parameters associated with TNF-α levels in univariate analysis.

Clinical Characteristics		TNF-α, pg/mL	*p*-Value
Age, years ^1^		*r* = −0.145	0.500
Sex ^2^	Male	0.9 (0.7–1.1)	1.000
	Female	1.0 (0.5–1.0)	
Hypertension ^2^	No	1.5 (1.1–1.5)	0.145
	Yes	0.9 (0.7–1.1)	
Dyslipidemia ^2^	No	1.5 (1.1–1.5)	0.181
	Yes	0.9 (0.7–1.1)	
Diabetes mellitus ^2^	No	0.9 (0.8–1.2)	0.820
	Yes	1.0 (0.6–1.3)	
Active smoking ^2^	No	0.9 (0.6–1.1)	0.347
	Yes	1.1 (0.8–1.3)	
Body mass index, kg/m^2 1^		*r* = 0.229	0.281
Number of coronary vessels with obstructive disease ^1,3^		*r* = 0.097	0.651
Prior CABG ^2^	No	1.0 (0.7–1.2)	0.742
	Yes	0.8 (0.6–0.8)	
Left ventricular ejection fraction ^2^	≤50%	–	–
	>50%	1.0 (0.7–1.1)	
Antiplatelet therapy ^2^	No	–	–
	Yes	1.0 (0.7–1.1)	
Oral anticoagulation ^2^	No	1.0 (0.7–1.1)	–
	Yes	–	
Statin therapy ^2^	No	0.8 (0.7–0.8)	0.587
	Yes	1.0 (0.7–1.2)	
High-intensity statin therapy ^2^	No	1.0 (0.8–1.2)	0.486
	Yes	0.9 (0.7–1.1)	
Ezetimibe ^2^	No	0.9 (0.7–1.1)	0.261
	Yes	1.1 (1.1–1.2)	
ACE inhibitor or ARB ^2^	No	0.8 (0.7–1.0)	0.431
	Yes	1.0 (0.7–1.3)	
Betablocker ^2^	No	0.9 (0.7–1.3)	0.861
	Yes	1.0 (0.7–1.1)	
Other antianginal agent ^2^	No	0.9 (0.6–1.1)	0.514
	Yes	1.0 (0.7–1.3)	
Oral antidiabetic agent ^2^	No	0.9 (0.8–1.2)	0.820
	Yes	1.0 (0.6–1.3)	
Insulin therapy ^2^	No	0.9 (0.7–1.0)	0.119
	Yes	1.3 (0.8–2.4)	
**Laboratory data** ^1^			
Hemoglobin, g/dL		*r* = -0.343	0.101
Leukocyte count, 10^9^/L		*r* = 0.167	0.436
Neutrophil count, 10^9^/L		*r* = 0.186	0.385
Lymphocyte count, 10^9^/L		*r* = -0.352	0.870
Platelet count, 10^9^/L		*r* = 0.195	0.360
Fasting glycaemia, mg/dL		*r* = 0.395	0.056
Percentage of glycosylated hemoglobin		*r* = 0.418	0.042
Creatinine, mg/dL		*r* = 0.362	0.082
Total cholesterol, mg/dL		*r* = 0.129	0.549
LDL-cholesterol, mg/dL		*r* = -0.094	0.662
HDL-cholesterol, mg/dL		*r* = -0.271	0.201
Triglycerides, mg/dL		*r* = 0.429	0.037
Soluble CD40 ligand, ng/mL		*r* = 0.170	0.427
C-reactive protein, mg/L		*r* = 0.407	0.048
**ΔC_t_ miRNA** ^1^			
miR-21		*r* = 0.278	0.199
miR-27b		*r* = 0.328	0.198
miR-29a		*r* = 0.189	0.627
miR-126		*r* = 0.374	0.139
miR-146a		*r* = 0.500	0.035
miR-218		*r* = 0.408	0.423

^1^ Correlations between TNF-α levels and continuous variables were tested and the correlation coefficient (r) is presented for each; ^2^ TNF-α levels were compared between groups for categorical variables and are expressed as the mean (standard deviation) or median (interquartile range); ^3^ the left main artery, left anterior descending artery, circumflex artery, and right coronary artery were scored individually. ACE—angiotensin-converting enzyme; ARB—angiotensin II receptor blocker; CABG—coronary artery bypass grafting; HDL—high-density lipoproteins; LDL—low-density lipoproteins; TNF-α—tumor necrosis factor alpha; ΔC_t_—delta cycle threshold.

**Table 3:** Parameters associated with TNF-α levels in multivariate analysis.

Parameters Associated with TNF-α Levels	β	95% CI	*p*-Value
Serum triglyceride levels	0.003	0.001–0.004	0.008
ΔC_t_ miR-146a	0.111	0.026–0.196	0.014

95% CI—95% confidence interval; TNF-α—tumor necrosis factor alpha; ΔC_t_—delta cycle threshold.

## Data Availability

The data presented in this study are available on request from the corresponding author. The data are not publicly available due to personal data protection.

## Supplementary Materials

The following are available online at https://www.mdpi.com/article/10.3390/medicina57060575/s1, Table S1: Clinical and laboratory parameters associated with miR-146a expression levels.

## References

[1] Virani S.S., Alonso A., Benjamin E.J., Bittencourt M.S., Callaway C.W., Carson A.P., Chamberlain A.M., Chang A.R., Cheng S., Delling F.N. (2020). Heart disease and stroke statistics-2020 update: A report from the American Heart Association. Circulation.

[2] Libby P., Ridker P.M., Hansson G.K., Atherothrombosis L.T.N.O. (2009). Inflammation in atherosclerosis: From pathophysiology to practice. J. Am. Coll. Cardiol..

[3] Pereira-da-Silva T., Napoleao P., Pinheiro T., Selas M., Silva F., Ferreira R.C., Carmo M.M. (2020). Inflammation is associated with the presence and severity of chronic coronary syndrome through soluble CD40 ligand. Am. J. Cardiovasc. Dis..

[4] Paffen E., DeMaat M.P. (2006). C-reactive protein in atherosclerosis: A causal factor?. Cardiovasc. Res..

[5] Rolski F., Błyszczuk P. (2020). Complexity of TNF-α signaling in heart disease. J. Clin. Med..

[6] Subirana I., Fitó M., Diaz O., Vila J., Francés A., Delpon E., Sanchis J., Elosua R., Muñoz-Aguayo D., Dégano I.R. (2018). Prediction of coronary disease incidence by biomarkers of inflammation, oxidation, and metabolism. Sci. Rep..

[7] Ridker P.M., Rifai N., Pfeffer M., Sacks F., Lepage S., Braunwald E. (2000). Elevation of tumor necrosis factor-alpha and increased risk of recurrent coronary events after myocardial infarction. Circulation.

[8] Ridker P.M., Everett B.M., Pradhan A., MacFadyen J.G., Solomon D.H., Zaharris E., Mam V., Hasan A., Rosenberg Y., Iturriaga E. (2019). Low-dose methotrexate for the prevention of atherosclerotic events. N. Engl. J. Med..

[9] Tardif J.C., Kouz S., Waters D.D., Bertrand O.F., Diaz R., Maggioni A.P., Pinto F.J., Ibrahim R., Gamra H., Kiwan G.S. (2019). Efficacy and safety of low-dose colchicine after myocardial infarction. N. Engl. J. Med..

[10] Padfield G.J., Din J.N., Koushiappi E., Mills N.L., Robinson S.D., Cruden N.e.M., Lucking A.J., Chia S., Harding S.A., Newby D.E. (2013). Cardiovascular effects of tumour necrosis factor α antagonism in patients with acute myocardial infarction: A first in human study. Heart.

[11] Gonzalez Y., Herrera M.T., Soldevila G., Garcia-Garcia L., Fabián G., Pérez-Armendariz E.M., Bobadilla K., Guzmán-Beltrán S., Sada E., Torres M. (2012). High glucose concentrations induce TNF-α production through the down-regulation of CD33 in primary human monocytes. BMC Immunol..

[12] Acharya P., Talahalli R.R. (2019). Aging and hyperglycemia intensify dyslipidemia-induced oxidative stress and inflammation in rats: Assessment of restorative potentials of ALA and EPA + DHA. Inflammation.

[13] Jonkers I.J., Mohrschladt M.F., Westendorp R.G., van der Laarse A., Smelt A.H. (2002). Severe hypertriglyceridemia with insulin resistance is associated with systemic inflammation: Reversal with bezafibrate therapy in a randomized controlled trial. Am. J. Med..

[14] Rosenson R.S., Davidson M.H., Hirsh B.J., Kathiresan S., Gaudet D. (2014). Genetics and causality of triglyceride-rich lipoproteins in atherosclerotic cardiovascular disease. J. Am. Coll. Cardiol..

[15] Breetha R., Ramaprasad T.R. (2018). Dietary n-3 but not n-6 fatty acids down-regulate maternal dyslipidemia induced inflammation: A three-generation study in rats. Prostaglandins Leukot Essent Fatty Acids.

[16] Bays H.E., Toth P.P., Kris-Etherton P.M., Abate N., Aronne L.J., Brown W.V., Gonzalez-Campoy J.M., Jones S.R., Kumar R., La Forge R. (2013). Obesity, adiposity, and dyslipidemia: A consensus statement from the National Lipid Association. J. Clin. Lipidol..

[17] Montazerifar F., Bolouri A., Mahmoudi Mozaffar M., Karajibani M. (2016). The prevalence of metabolic syndrome in coronary artery disease patients. Cardiol. Res..

[18] Sanada T., Sano T., Sotomaru Y., Alshargabi R., Yamawaki Y., Yamashita A., Matsunaga H., Iwashita M., Shinjo T., Kanematsu T. (2020). Anti-inflammatory effects of miRNA-146a induced in adipose and periodontal tissues. Biochem. Biophys. Rep..

[19] Feinberg M.W., Moore K.J. (2016). MicroRNA regulation of atherosclerosis. Circ. Res..

[20] Ridker P.M., Everett B.M., Thuren T., MacFadyen J.G., Chang W.H., Ballantyne C., Fonseca F., Nicolau J., Koenig W., Anker S.D. (2017). Antiinflammatory therapy with canakinumab for atherosclerotic disease. N. Engl. J. Med..

[21] Andreou I., Sun X., Stone P.H., Edelman E.R., Feinberg M.W. (2015). miRNAs in atherosclerotic plaque initiation, progression, and rupture. Trends Mol. Med..

[22] Chen L.J., Lim S.H., Yeh Y.T., Lien S.C., Chiu J.J. (2012). Roles of microRNAs in atherosclerosis and restenosis. J. Biomed. Sci..

[23] Pereira-da-Silva T., Coutinho Cruz M., Carrusca C., Cruz Ferreira R., Napoleão P., Mota Carmo M. (2018). Circulating microRNA profiles in different arterial territories of stable atherosclerotic disease: A systematic review. Am. J. Cardiovasc. Dis..

[24] Navickas R., Gal D., Laucevičius A., Taparauskaitė A., Zdanytė M., Holvoet P. (2016). Identifying circulating microRNAs as biomarkers of cardiovascular disease: A systematic review. Cardiovasc. Res..

[25] Kumar D., Narang R., Sreenivas V., Rastogi V., Bhatia J., Saluja D., Srivastava K. (2020). Circulatory miR-133b and miR-21 as novel biomarkers in early prediction and diagnosis of coronary artery disease. Genes.

[26] Zhang L., Zhang Y., Xue S., Ding H., Wang Y., Qi H., Zhu W., Li P. (2020). Clinical significance of circulating microRNAs as diagnostic biomarkers for coronary artery disease. J. Cell Mol. Med..

[27] Fichtlscherer S., De Rosa S., Fox H., Schwietz T., Fischer A., Liebetrau C., Weber M., Hamm C.W., Röxe T., Müller-Ardogan M. (2010). Circulating microRNAs in patients with coronary artery disease. Circ. Res..

[28] Malik R., Mushtaque R.S., Siddiqui U.A., Younus A., Aziz M.A., Humayun C., Mansoor K., Latif M.A., Waheed S., Assad S. (2017). Association between coronary artery disease and MicroRNA: Literature review and clinical perspective. Cureus.

[29] Xu Z., Han Y., Liu J., Jiang F., Hu H., Wang Y., Liu Q., Gong Y., Li X. (2015). MiR-135b-5p and MiR-499a-3p promote cell proliferation and migration in atherosclerosis by directly targeting MEF2C. Sci. Rep..

[30] Deo A., Carlsson J., Lindlöf A. (2011). How to choose a normalization strategy for miRNA quantitative real-time (qPCR) arrays. J. Bioinform. Comput. Biol..

[31] Wolfinger R.D., Beedanagari S., Boitier E., Chen T., Couttet P., Ellinger-Ziegelbauer H., Guillemain G., Mariet C., Mouritzen P., O’Lone R. (2018). Two approaches for estimating the lower limit of quantitation (LLOQ) of microRNA levels assayed as exploratory biomarkers by RT-qPCR. BMC Biotechnol..

[32] Zhang X., Shao S., Geng H., Yu Y., Wang C., Liu Z., Yu C., Jiang X., Deng Y., Gao L. (2014). Expression profiles of six circulating microRNAs critical to atherosclerosis in patients with subclinical hypothyroidism: A clinical study. J. Clin. Endocrinol. Metab..

[33] Vegter E.L., Ovchinnikova E.S., van Veldhuisen D.J., Jaarsma T., Berezikov E., van der Meer P., Voors A.A. (2017). Low circulating microRNA levels in heart failure patients are associated with atherosclerotic disease and cardiovascular-related rehospitalizations. Clin. Res. Cardiol..

[34] Stather P.W., Sylvius N., Sidloff D.A., Dattani N., Verissimo A., Wild J.B., Butt H.Z., Choke E., Sayers R.D., Bown M.J. (2015). Identification of microRNAs associated with abdominal aortic aneurysms and peripheral arterial disease. Br. J. Surg..

[35] Huang Y.Q., Li J., Chen J.Y., Zhou Y.L., Cai A.P., Huang C., Feng Y.Q. (2017). The association of circulating MiR-29b and Interleukin-6 with subclinical atherosclerosis. Cell Physiol. Biochem..

[36] Hansson G.K. (2005). Inflammation, atherosclerosis, and coronary artery disease. N. Engl. J. Med..

[37] Ciaraldi T.P., Carter L., Mudaliar S., Kern P.A., Henry R.R. (1998). Effects of tumor necrosis factor-alpha on glucose metabolism in cultured human muscle cells from nondiabetic and type 2 diabetic subjects. Endocrinology.

[38] Feingold K.R., Grunfeld C. (1987). Tumor necrosis factor-alpha stimulates hepatic lipogenesis in the rat in vivo. J. Clin. Investig..

[39] Zhou C., Zhao L., Wang K., Qi Q., Wang M., Yang L., Sun P., Mu H. (2019). MicroRNA-146a inhibits NF-κB activation and pro-inflammatory cytokine production by regulating IRAK1 expression in THP-1 cells. Exp. Ther. Med..

[40] Chen B.B., Li Z.H., Gao S. (2018). Circulating miR-146a/b correlates with inflammatory cytokines in COPD and could predict the risk of acute exacerbation COPD. Medicine (Baltimore).

[41] Feng Y., Chen L., Luo Q., Wu M., Chen Y., Shi X. (2018). Involvement of microRNA-146a in diabetic peripheral neuropathy through the regulation of inflammation. Drug Des. Devel. Ther..

[42] Pereira-da-Silva T., Napoleão P., Costa M.C., Gabriel A.F., Selas M., Silva F., Enguita F.J., Ferreira R.C., Carmo M.M. (2021). Circulating miRNAs are associated with the systemic extent of atherosclerosis: Novel observations for miR-27b and miR-146. Diagnostics.

[43] Cheng H.S., Sivachandran N., Lau A., Boudreau E., Zhao J.L., Baltimore D., Delgado-Olguin P., Cybulsky M.I., Fish J.E. (2013). MicroRNA-146 represses endothelial activation by inhibiting pro-inflammatory pathways. EMBO Mol. Med..

[44] Li K., Ching D., Luk F.S., Raffai R.L. (2015). Apolipoprotein E enhances microRNA-146a in monocytes and macrophages to suppress nuclear factor-κB-driven inflammation and atherosclerosis. Circ. Res..

[45] Yang K., He Y.S., Wang X.Q., Lu L., Chen Q.J., Liu J., Sun Z., Shen W.F. (2011). MiR-146a inhibits oxidized low-density lipoprotein-induced lipid accumulation and inflammatory response via targeting toll-like receptor 4. FEBS Lett..

[46] Hanna J., Hossain G.S., Kocerha J. (2019). The potential for microRNA therapeutics and clinical research. Front. Genet..

[47] Sabbatinelli J., Giuliani A., Matacchione G., Latini S., Laprovitera N., Pomponio G., Ferrarini A., Svegliati Baroni S., Pavani M., Moretti M. (2021). Decreased serum levels of the inflammaging marker miR-146a are associated with clinical non-response to tocilizumab in COVID-19 patients. Mech. Ageing Dev..

