# Supplementary material for: Association between miR-146a and Tumor Necrosis Factor Alpha (TNF-α) in Stable Coronary Artery Disease
Source: Medicina (Kaunas). 2021 Jun 4;57(6):575. doi: 10.3390/medicina57060575 (PMC8230353; doi:10.3390/medicina57060575)
Supplement: Supplementary file 1 [file medicina-57-00575-s001.zip › medicina-1250942-supplementary.pdf]

Table S1. Clinical and laboratory parameters associated with miR-146a expression levels.

		ΔC_t miR-146a	*p*-value
**Clinical characteristics**			
Age, years [1]		*r* = 0.085	0.738
Sex [2]	Male	22.2 (2.4)	0.117
	Female	25.1 (2.1)	
Hypertension [2]	No	21.6 (2.5)	0.711
	Yes	22.6 (2.5)	
Dyslipidemia [2]	No	21.8 (2.4)	0.701
	Yes	22.5 (2.5)	
Diabetes mellitus [2]	No	22.4 (2.1)	0.827
	Yes	22.6 (2.8)	
Active smoking [2]	No	22.6 (2.6)	0.865
	Yes	22.3 (2.2)	
Body mass index, kg/m^2 [1]		*r* = -0.181	0.472
Number of coronary vessels with obstructive disease [1,3]		*r* = 0.556	0.017
Prior CABG [2]	No	22.9 (2.5)	0.097
	Yes	20.3 (1.1)	
Left ventricular ejection fraction [2]	≤ 50%	–	–
	> 50%	22.5 (2.5)	
Antiplatelet therapy [2]	No	–	–
	Yes	22.5 (2.5)	
Oral anticoagulation [2]	No	22.5 (2.5)	–
	Yes	–	
Statin therapy [2]	No	21.3 (2.5)	0.618
	Yes	22.6 (2.5)	
High-intensity statin therapy [2]	No	23.0 (2.3)	0.426
	Yes	22.0 (2.7)	
Ezetimibe [2]	No	22.6 (2.6)	0.750
	Yes	22.0 (0.5)	
ACE inhibitor or ARB [2]	No	22.7 (2.2)	0.895
	Yes	22.5 (2.6)	
Betablocker [2]	No	23.4 (1.7)	0.136
	Yes	21.6 (2.9)	
Other antianginal agent [2]	No	21.9 (2.8)	0.138
	Yes	23.8 (1.1)	
Oral antidiabetic agent [2]	No	22.4 (2.1)	0.825
	Yes	22.6 (2.8)	
Insulin therapy [2]	No	22.5 (2.2)	0.984
	Yes	22.5 (3.7)	
**Laboratory data** [1]			
Hemoglobin, g/dL		*r* = -0.488	0.040
Leukocyte count, $10^9/L$		*r* = -0.090	0.723
Neutrophil count, $10^9/L$		*r* = -0.270	0.279
Lymphocyte count, $10^9/L$		*r* = 0.271	0.277
Platelet count, $10^9/L$		*r* = 0.263	0.292
Fasting glycaemia, mg/dL		*r* = 0.371	0.129
Percentage of glycosylated hemoglobin		*r* = 0.317	0.200

Creatinine, mg/dL	*r* = 0.337	0.171
Total cholesterol, mg/dL	*r* = 0.280	0.260
LDL-cholesterol, mg/dL	*r* = -0.058	0.820
HDL-cholesterol, mg/dL	*r* = 0.294	0.236
Triglycerides, mg/dL	*r* = 0.029	0.908
Soluble CD40 ligand, ng/mL	*r* = 0.312	0.236
C-reactive protein, mg/L	*r* = 0.304	0.207

[1] Correlations between ΔC_t miR-146a and continuous variables were tested and the correlation coefficient (r) is presented for each; [2] ΔC_t miR-146a was compared between groups for categorical variables and is expressed as the mean (standard deviation) or median (interquartile range); [3] the left main artery, left anterior descending artery, circumflex artery, and right coronary artery were scored individually. ACE – angiotensin-converting enzyme; ARB – angiotensin II receptor blocker; CABG – coronary artery bypass grafting; HDL – high-density lipoproteins; LDL – low-density lipoproteins; ΔC_t – delta cycle threshold.
